# Supplementary material for: Dynamic imaging of crystalline defects in lithium-manganese oxide electrodes during electrochemical activation to high voltage
Source: Nat Commun. 2019 Apr 12;10:1692. doi: 10.1038/s41467-019-09408-2 (PMC6461632; doi:10.1038/s41467-019-09408-2)
Supplement: Supplementary file 1 — Supplementary Information [file 41467_2019_9408_MOESM1_ESM.pdf]

Supplementary Information for

Dynamic imaging of crystalline defects in lithium manganese oxide electrodes during electrochemical activation to high voltage

Qianqian Li[23†*], Zhenpeng Yao[3z†], Eungje Lee[4], Yaobin Xu[3], Michael M. Thackeray[4], Chris Wolverton[3], Vinayak P. Dravid[3*] & Jinsong Wu[13*]

[1] State Key Laboratory of Advanced Technology for Materials Synthesis and Processing, Wuhan University of Technology, Wuhan, Hubei, China

[2] Materials Genome Institute, Shanghai University, Shanghai, China

[3] Department of Materials Science and Engineering, NU*ANCE* Center, Northwestern University, Evanston, Illinois 60208, USA

[4] Chemical Sciences and Engineering Division, Argonne National Laboratory, Argonne, Illinois 60439, USA

[†]These authors contributed equally to this work.

[z]Current Address: Department of Chemistry and Chemical Biology, Harvard University, 12 Oxford Street, Cambridge, Massachusetts 02138, United States

*E-mail: wujs@whut.edu.cn; v-dravid@northwestern.edu; qianqianli@shu.edu.cn

1. Supplementary Figures

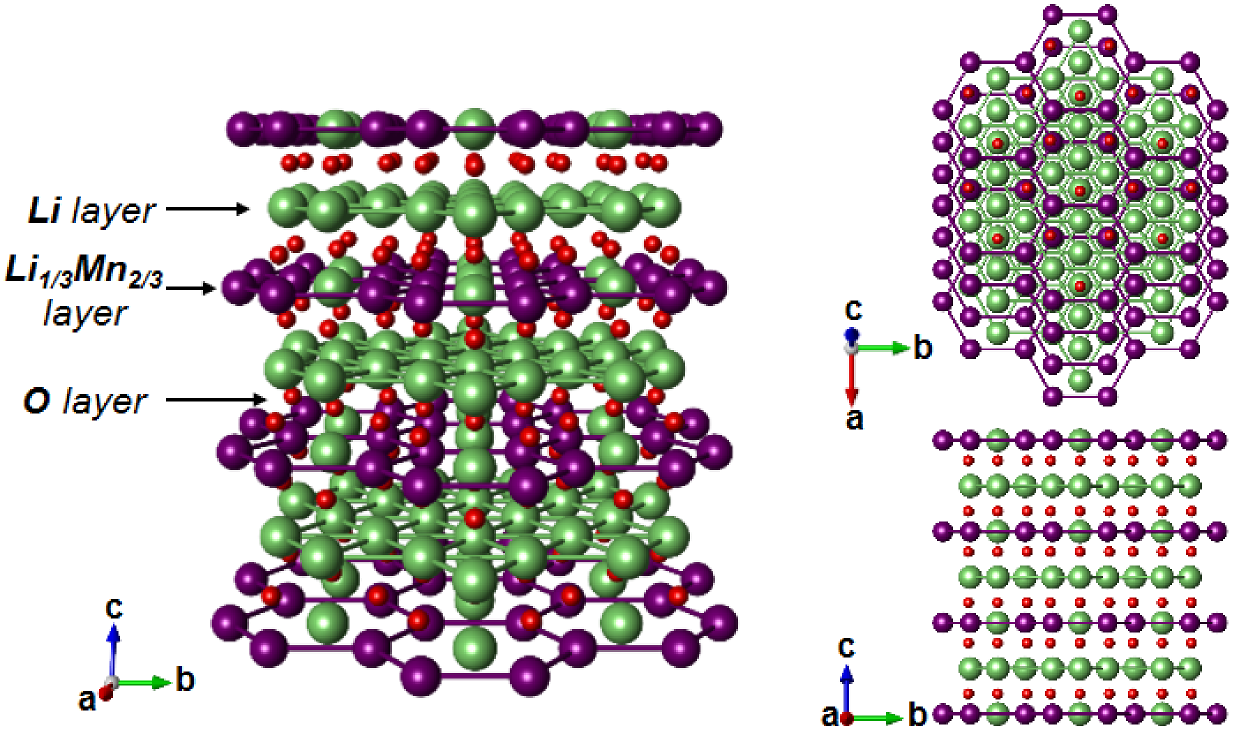

Supplementary Figure 1. Illustration of atomic structure of Li_2MnO_3. Li_2MnO_3 has a monoclinic lattice (space group of *C2/m*) in which Li^+ and Mn^{4+} ions reside in the octahedral sites of a cubic close packed oxygen lattice. The structure consists of alternating Li layers and Li/Mn (in a 1/2 ratio) layers along the [001] direction and can be denoted as $Li[Li_{1/3}Mn_{2/3}]O_2$. The stacking of the $Li[Li_{1/3}Mn_{2/3}]O_2$ layers is ABC_1 in the structure free of planar defects.

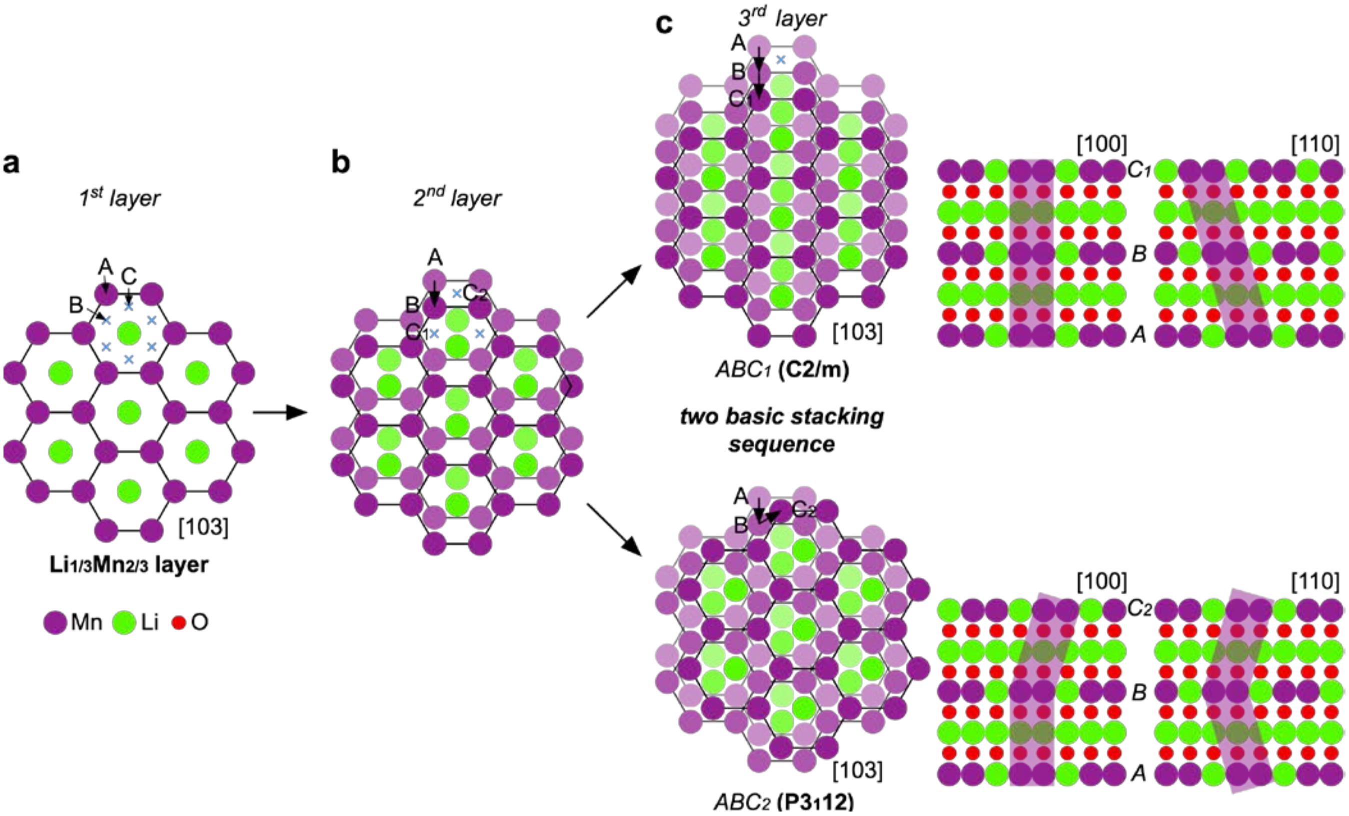

Supplementary Figure 2. The stacking of the honey-comb $Li_{1/3}Mn_{2/3}$ layers in Li_2MnO_3 and the origin of honey-comb layer stacking faults. (a) One honey-comb layer with purple circles, green circles, and blue crosses stand for Mn ions, Li ions, and metal sites to realize close-packing between layers, respectively. (b) The single way to add the second honey-comb layer. (c) Two basic stacking sequences: original stacking (ABC_1) as in Li_2MnO_3 (space group: C2/m) and stacking fault (ABC_2, space group: $P3_112$) with corresponding Mn patterns as observed from [100] and [110] directions.

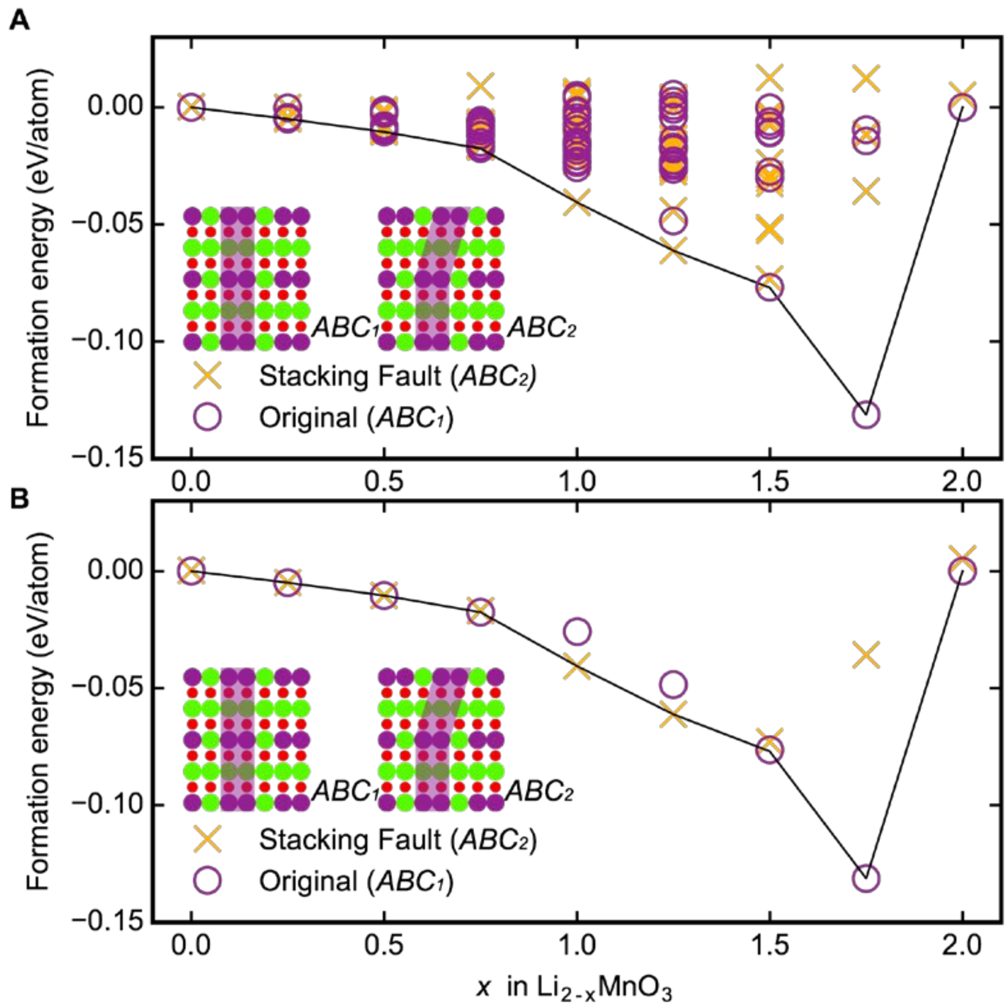

Supplementary Figure 3. Delithiation convex hull of Li_2MnO_3 with original stacking order (ABC_1) and stacking fault (ABC_2).

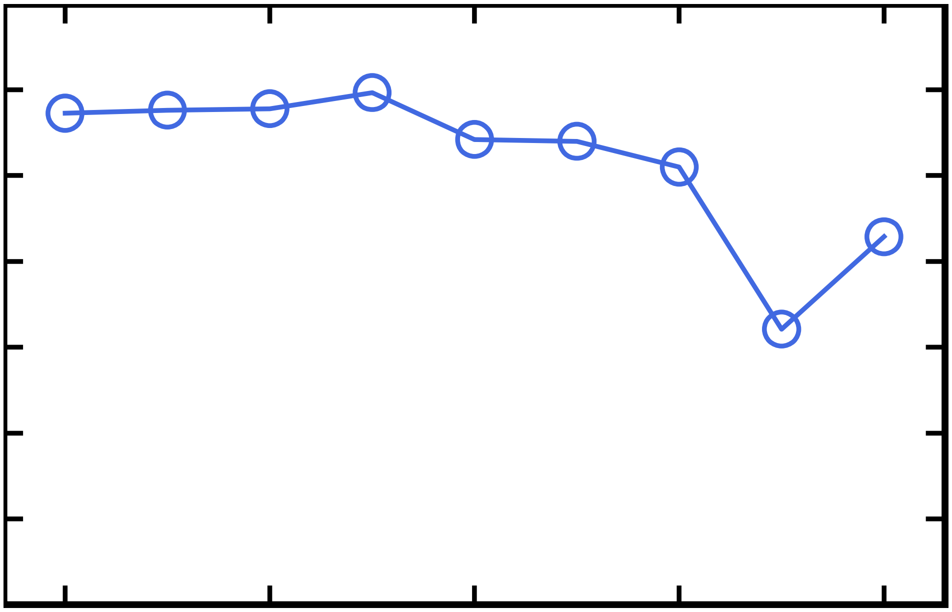

Supplementary Figure 4. The plot of calculated (001) *d*-spacing of delithiated Li_2MnO_3. The *d*-spacing is gradually increased when the *x* is smaller than ~0.75. It is decreased afterward when more lithium ions have been extracted.

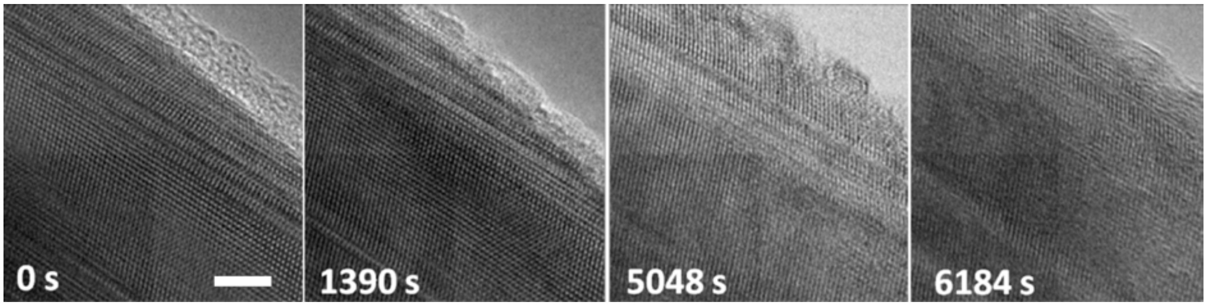

Supplementary Figure 5. The evolution of (001) stacking fault with fault vector of *b*/6[110] in delithiation observed by in-situ TEM. The time clasped HREM images showing the change of the (001) lattice plane stacking along with the delithiation process. Scale bar: 5nm.

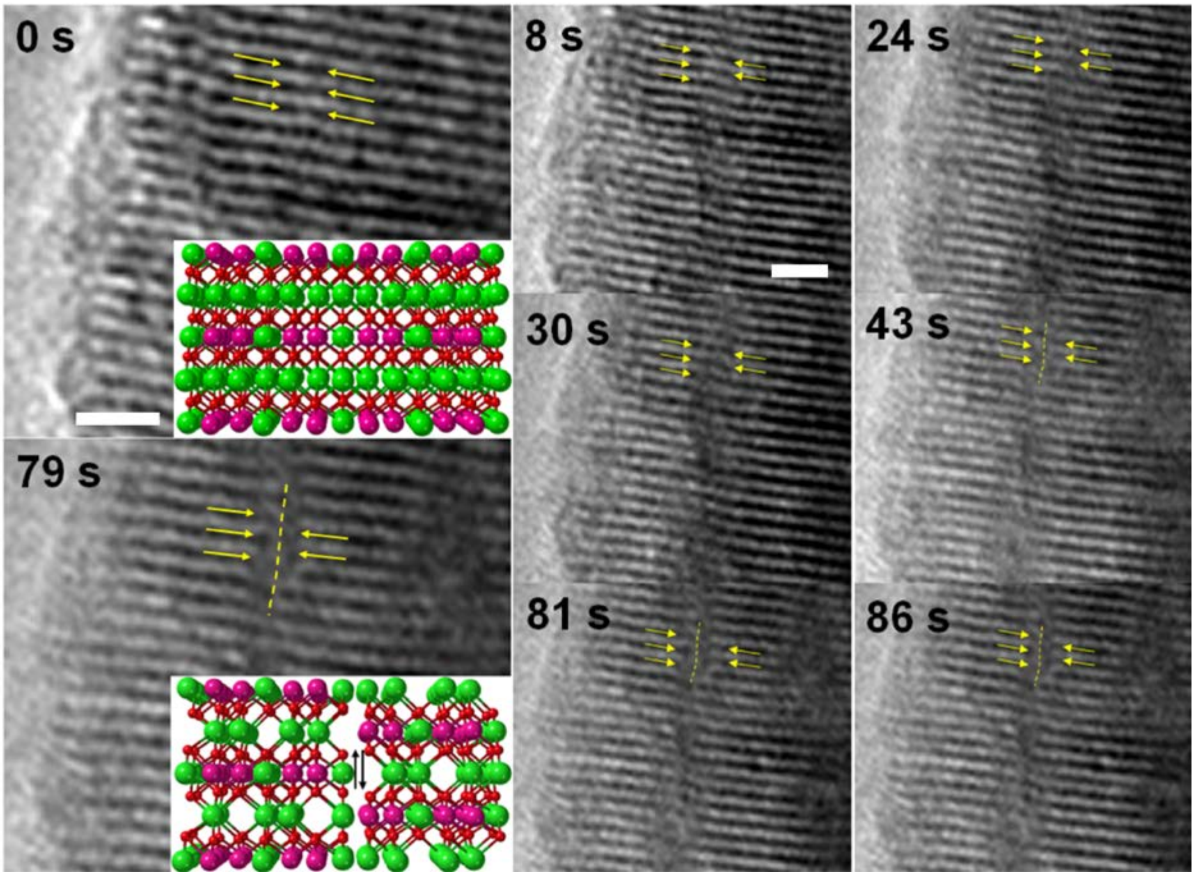

Supplementary Figure 6. A dissociated dislocation with Burgers vector of *c*/2[001] formed dynamically in delithiation. The time clasped HREM images (from 0 to 86 second) showing the formation of the dislocation along with the delithiation. The atomic models of a pre-delithiated Li_2MnO_3 and a delithiated Li_2MnO_3 with the dislocation are shown as inserts. Scale bar: 2nm.

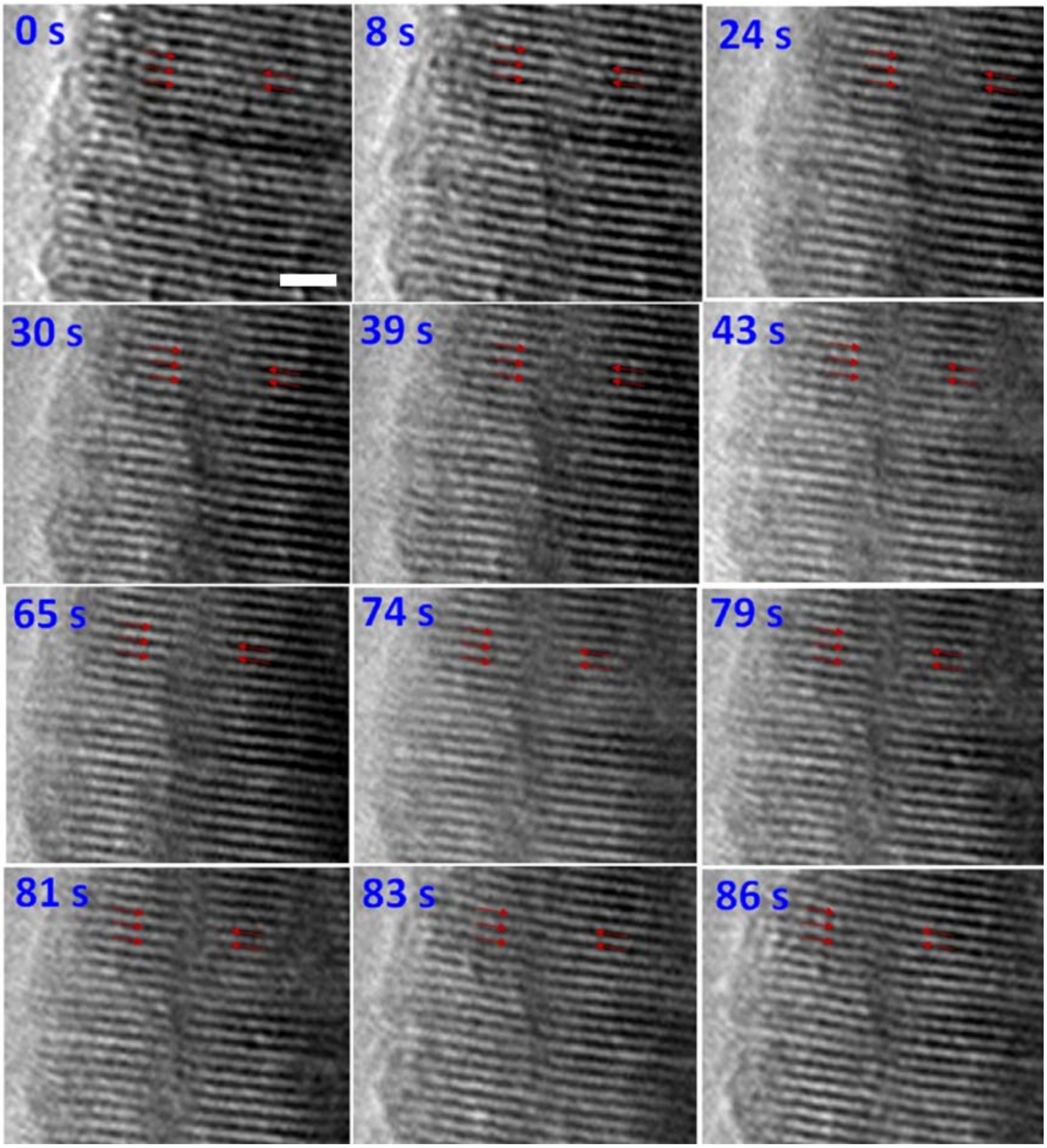

Supplementary Figure 7. The time clasped HREM images corresponding to generation of the *c*/2[001] dislocation formed dynamically in delithiation. Scale bar: 2nm.

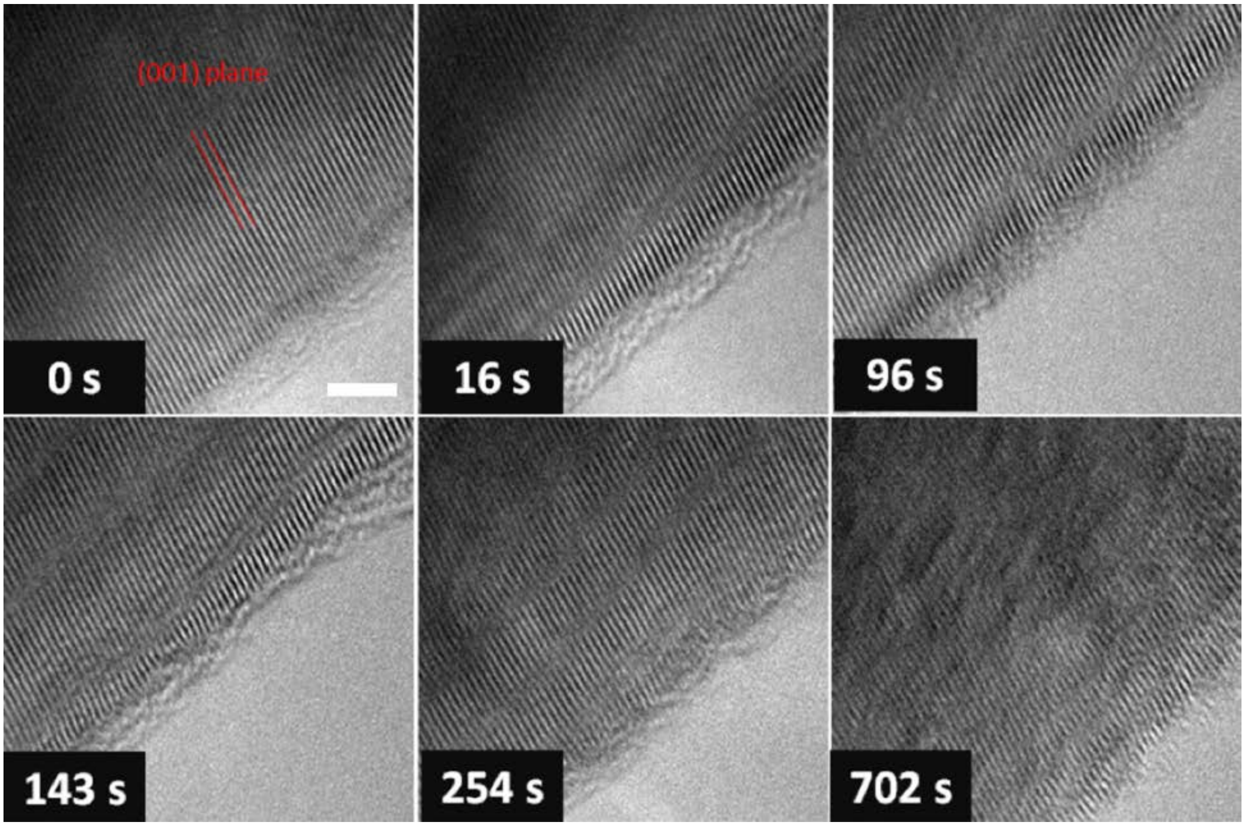

Supplementary Figure 8. The time clasped HREM images corresponding to generation of the *c*/2[001] dislocation formed dynamically in delithiation. The defect cuts the large Li_2MnO_3 crystal into small ones. The density of the defects increased along with the delithiation process. Scale bar: 5nm.

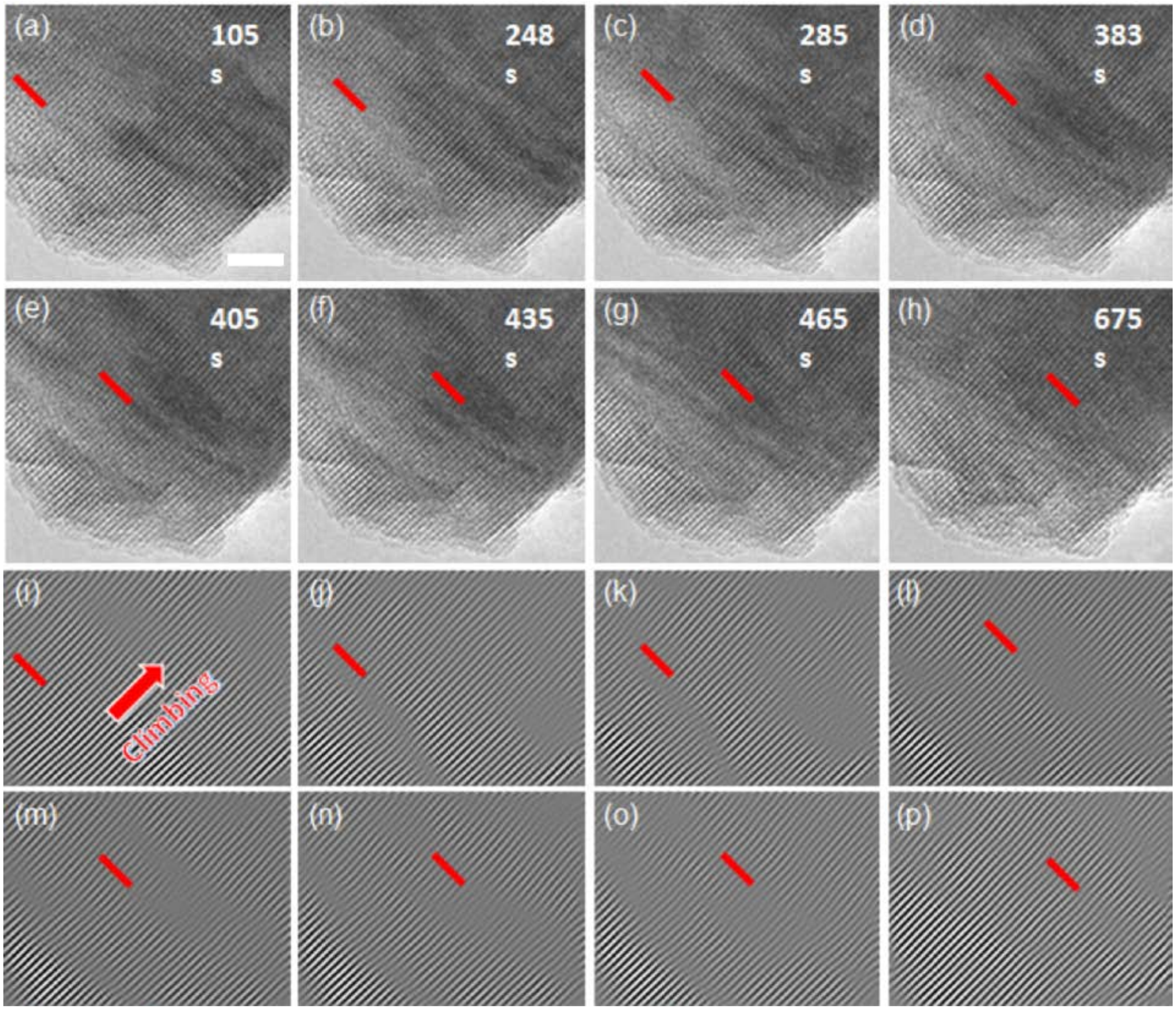

Supplementary Figure 9. The dynamic climbing of *c*/2[001] dislocation in delithiation. (a)-(h) time clasped HREM images of the evolution of c/2[001] dislocations in Li_2MnO_3 during delithiation. (i)-(p) The corresponding Fourier filtered images of (a)-(h), showing only the (001) lattice plane fringes. Scale bar: 5nm.

3. Supplementary Notes

3.1. Stacking ordering

Li_2MnO_3, which can also be written as $Li[Li_{1/3}Mn_{2/3}]O_2$, adopts a layered structure constituted by alternative "lithium" (Li) layers and "transition metal" ($Li_{1/3}Mn_{2/3}$) layers (Supplementary Figure 1). In the "transition metal" layer, Li and Mn ions arranged in a "honey comb" scheme with a ratio of 1:2 (Supplementary Figure 2a). Different stacking ordering of the transition metal ($Li_{1/3}Mn_{2/3}$) layers then can introduce distinct crystal symmetries to the system. As shown in the Supplementary Figure 2b, the addition of the second "transition metal" layer will inevitably generate an AB stacking to achieve a "close-packed" array. Yet there are two possibilities for the addition of the third layer, which are ABA and ABC orderings, corresponding to two basic stacking sequences: C2/m (ABC_1) and $P3_112$ (ABC_2). More complex stacking orderings (*e.g.* C2/c)[1] therefore can be achieved by the combination of these two basic sequences. Here in this study, we thus start with these two basic stacking sequences to investigate the stacking ordering evolution during the delithiation process. The Non-equilibrium Phase Search (NEPS) method[2,3] was applied by exploring the symmetrically distinct Li/vacancy configurations on Li sites of Li_2MnO_3 with these two basic sequences as a function of Li concentration (Li/vacancy ratios). The method proceeds as follows: (i) Create supercells of Li_2MnO_3 with two basic stacking sequences: original C2/m (ABC_1) and stacking fault $P3_112$ (ABC_2) which contains 8 Li^+ ions, 4 Mn^{4+} ions, and 6 O^{2-} ions. (ii) Generate all symmetrically distinct configurations for both two stacking sequences using Enum (Enum is the name of an algorithm/code for enumerating/generating all derivative superstructures for arbitrary parent structures and for any number of atom types) for a series of compositions $Li_{2-x}\square_x MnO_3$ ($0 < x < 2$, $\square$ denoting vacancy). (iii) Sample the total energies of all configurations using DFT with

settings described in Section below. (iv) For the specific composition, formation energies of the configurations corresponding to the two stacking sequences are calculated according to following reaction: $Li_2MnO_3 \rightarrow x$Li + $Li_{2-x}\square_xMnO_3$. (v) Track the stacking fault evolution by checking the structures of the lowest energy configurations during delithiation (Supplementary Figure 3).

As shown in Supplementary Figure 3, in the intact Li_2MnO_3 and initial delithiation period ($Li_{2-x}MnO_3$, $0 < x < 0.75$), lowest energy configurations from both stacking sequences show extremely close formation energies, indicating the co-existence of those basic stacking sequences and also their combinations as observed in many previous studies.[4,5] The Li removal occurs primarily *via* extracting the Li from the "lithium" layer. In the subsequent delithiation period ($Li_{2-x}MnO_3$, $0.75 < x < 1.5$), there is a stacking sequence transition from original (ABC_1) to stacking fault (ABC_2) and then back to the original (ABC_1). In this stage, Li ions start to be removed from the "transition metal" layers along with the "lithium" layers. In the final delithiation period ($Li_{2-x}MnO_3$, $1.5 < x < 2.0$), the original ABC_1 stacking sequence is predicted to be dominant. The "lithium" layers are depleted followed by the completion of the delithiation. The calculated voltages (Fig.2g) exhibit two steps with the first one corresponding to the pure Li removal and matches the 4.5-5.0 V slope as observed in the experimental charge.[6] A higher voltage around 5.13 V (Fig.2g) is predicted to be necessary to enable the oxygen extraction, which is higher than what observed in the experimental test and suggests the contribution of other mechanisms as discussed in the following Section.

The oxygen releasing is also monitored by evaluating the O^0 vacancy formation energy as a function of Li removal with a lower $\Delta E_{\mathrm{Vac}}^{Form}$ implying more facile O extractions and a negative $\Delta E_{\mathrm{Vac}}^{Form}$ implying the spontaneous oxygen releasing. As shown in Fig. 4a, the oxygen vacancy

formation energy keeps decreasing during the Li removal. However, the oxygen vacancy formation energy stays positive in a wide Li concentration range (0.0 < x < 1.5) and the Li removal is charge compensated by the partial oxygen oxidation ($O^{2-} \rightarrow O^{1-}$). The spontaneous oxygen releasing ($O^{2(1)-} \rightarrow O^{0}$) only occurs after a large amount of Li ions have been extracted from the system, *i.e.* the final delithiation period ($Li_{2-x}MnO_3$, $x > 1.5$). It is noteworthy that, the spontaneous oxygen releasing occurs at the same time then the depletion of the “lithium” layers, which can be explained by the electrostatically unfavored direct confrontment of oxygen ions and the significantly shortened O-O distance from 3.2 Å to 1.5 Å and 2.2 Å (Fig.4c) after the complete Li extraction between oxygen layers.

3.2 *c*/2[001] dislocation

To study the energetic impact of the *c*/2[001] dislocation, as formed during the delithiation process, we built supercells with the dislocations for selected compositions during the delithiation $Li_{2-x}MnO_3$ (x = 0, 1, 2) which contains 32 Li^{+} ions, 16 Mn^{4+} ions and 48 O^{2-} ions (x = 0). The dislocations are introduced through shifting the atom positions of half of the ions by1/2[001] in the *c* direction from the original configurations as identified above. We first access the tendency of the *c*/2[001] dislocation formation during the delithiation by calculating the energy difference between the configurations with and without *c*/2[001] dislocation introduced. As shown in Fig.4b, the energy difference decreases from 0.39 eV/atom (x = 0) to 0.06 eV/atom (x = 1), and becomes negative at x = 2. In reality, Li depletion is localized (e.g. areas on the particle surface) which can facilitate the formations of the c/2 dislocation. Oxygen vacancy formation energies are also calculated in systems with *c*/2[001] dislocation introduced in order to estimate the impact of dislocations on the oxygen releasing. As shown in Fig.4a, oxygen vacancy formation energies are greatly decreased in the systems with *c*/2[001] dislocation compared to

the original systems for all the selected compositions ($Li_{2-x}MnO_3$, $x = 0, 1, 2$), respectively. And the spontaneous oxygen releasing is brought forward to around $x = 1$, compared to $x > 1.5$ for the system without *c*/2[001] dislocation. As discussed in the previous Section, the spontaneous oxygen releasing can be understood as the results of the electrostatically unfavored distance decrease between the oxygen ions. When introducing the *c*/2[001] dislocation to the system, an interphase is created with the oxygen ions at the interface experience a significant distance decrease from 3.2 Å to 1.6 Å (Fig.4d). As a result, oxygen releasing becomes facile without the requirement of a large amount of Li removal. And the voltage plateau corresponding to oxygen releasing is lowered to be around 5.03 V, well matched what observed during the experimental charge of Li_2MnO_3.[6] Furthermore, since the dislocations are observed to be climbing towards the surface upon delithiation, the O^0 removed from the inside of the particle then can be shipped to its surface. Therefore, the *c*/2[001] dislocation assisted oxygen releasing from Li_2MnO_3 proceeds as follows (Fig.2h): (i) Formation of dislocations after a certain amount of Li extraction throughout the particle. (ii) O^0 vacancies formed at the dislocations with the extracted O^0 stayed at the interphase. (iii) *c*/2[001] dislocation climbing with O^0 being transported to the surface. (iv) O^0 atoms gathered together at the surface and form the O_2 molecules.

4. Supplementary Methods

4.1. DFT calculations

DFT calculations reported in this study were conducted *via* the Vienna Ab-initio Simulation Package (VASP)[7–10] with the projector augmented wave (PAW) potentials and the Perdew-Becke-Ernzerhof (PBE) approximation was employed to the exchange-correlation potential. A plane wave basis with a cutoff energy of 520 eV and ***Γ***-centered *k*-meshes with a density of 8000 *k*-points per reciprocal atom were used for all calculations. All calculations were spin-polarized, with Mn atoms initialized in a high-spin configuration and relaxed to self-consistency. The DFT + *U* method introduced by Dudarev *et al.*[11] was used to treat the localized 3*d* electrons of Mn with a *U* value of 3.8, obtained by fitting it to experimental and calculated formation enthalpies in a previous study.[12] Heyd-Scuseria-Ernzerhof screened hybrid functional (HSE06) was used to accurately determine the energies, magnetic and electronic states of Mn and O in the delithiated phases with structures relaxed using DFT + *U*: $Li_{2-x}Mn_2O_3$ ($0 < x < 2$).

4.2. Voltage profile calculation

Considering a two-phase reaction between Li_xMnO_3 and Li_yMnO_3: $Li_xMnO_3 + (y - x)Li \rightarrow Li_yMnO_3$, the average delithiation voltage (versus Li/Li^+) is given by the negative of the reaction free energy per Li removed, as shown in Eq. (1):[13]

$$V = \frac{\Delta G_{\mathrm{f}}}{F \Delta N_{\mathrm{Li}}} \quad \text{(S1)}$$

where ΔG_{f} is the (molar) change in free energy of the reaction, ΔN_{Li} is the amount of Li removed, and F is the Faraday constant. ΔG_{f} can be approximated by the total internal energies from DFT calculations neglecting the entropic contributions (0 K):

$$\Delta G_{\mathrm{f}} \sim \Delta E = E\left(\mathrm{Li}_y\mathrm{MnO}_3\right) - E(\mathrm{Li}_x\mathrm{MnO}_3) - (y - x)E(\mathrm{Li}_{\mathrm{metal}}) \quad \text{(S2)}$$

where $E(\mathrm{Li}_y\mathrm{MnO}_3)$ and $E(\mathrm{Li}_x\mathrm{MnO}_3)$ are the DFT energies at the corresponding compositions.

4.3. Oxygen releasing evaluation

Oxygen releasing during the delithiation process of Li_2MnO_3 is monitored by calculating the oxygen vacancy (O^0) formation energy as a function of Li removal. The oxygen vacancy formation energy can be evaluated as shown in Eq. S3:

$$\Delta E_{\mathrm{Vac}}^{Form} = E_{\mathrm{Vac}}(\mathrm{Li}_{2\text{-}x}\mathrm{MnO}_{3\text{-}\delta}) - E(\mathrm{Li}_{2\text{-}x}\mathrm{MnO}_3) - \sum n\mu_{\mathrm{O}} \quad \text{(S3)}$$

where $E_{\mathrm{Vac}}(\mathrm{Li}_{2\text{-}x}\mathrm{MnO}_{3\text{-}\delta})$ is the energy of a supercell with a single O^0 vacancy at a specific Li concentration and $E(\mathrm{Li}_{2\text{-}x}\mathrm{MnO}_3)$ is the energy of a supercell without any defect at the same Li concentration. When creating the defect $\mathrm{Li}_{2\text{-}x}\mathrm{MnO}_{3\text{-}\delta}$ structure, the O^0 vacancy was introduced into the original $\mathrm{Li}_{2\text{-}x}\mathrm{MnO}_3$ structure by removing the O ion which experiences the largest valence variation during the Li removal. The valence variation was evaluated by monitoring the O magnetic moment changes following previous studies.[14,15] $\sum n\mu_{\mathrm{O}}$ is the total chemical potential of the oxygen vacancy; μ_{O} is the chemical potential of O^0 atom and n is the number of O^0 atom when introducing oxygen vacancy ($n = 1$). The chemical potential of O^0 was fitted[16] to experiment data from SGTE substance database (SSUB).[17]

5. Supplementary References

1. Riou, A., Lecerf, A., Gerault, Y. & Cudennec, Y. Etude structurale de Li2MnO3. *Mater. Res. Bull.* **27**, 269–275 (1992).
2. Yao, Z. *et al.* Revealing the Conversion Mechanism of Transition Metal Oxide Electrodes during Lithiation from First-Principles. *Chem. Mater.* **29**, 9011–9022 (2017).
3. Li, Q. *et al.* Dynamic imaging of metastable reaction pathways in lithiated metal oxide electrodes. *Nano Energy* **44**, 15–22 (2017).
4. Wang, R. *et al.* Atomic structure of Li_2MnO_3 after partial delithiation and re-lithiation.

Adv. Energy Mater. **3**, 1358–1367 (2013).

5. Boulineau, A., Croguennec, L., Delmas, C. & Weill, F. Reinvestigation of Li2MnO3 Structure: Electron Diffraction and High Resolution TEM. *Chem. Mater.* **21**, 4216–4222 (2009).

6. Thackeray, M. M. *et al.* Li2MnO3-stabilized LiMO2 (M = Mn, Ni, Co) electrodes for lithium-ion batteries. *J. Mater. Chem.* **17**, 3112–3125 (2007).

7. Kresse, G. & Hafner, J. Ab Initio Molecular Dynamics for Liquid Metals. *Phys. Rev. B* **47**, 558–561 (1993).

8. Kresse, G. & Hafner, J. Ab Initio Molecular-dynamics Simulation of the Liquid-metal-amorphous-semiconductor Transition in Germanium. *Phys. Rev. B* **49**, 14251–14269 (1994).

9. Kresse, G. & Furthmüller, J. Efficiency of Ab-initio Total Energy Calculations for Metals and Semiconductors Using a Plane-wave Basis Set. *Comput. Mater. Sci.* **6**, 15–50 (1996).

10. Kresse, G. Efficient Iterative Schemes for Ab Initio Total-energy Calculations Using a Plane-Wave Basis Set. *Phys. Rev. B* **54**, 11169–11186 (1996).

11. Dudarev, S. L., Savrasov, S. Y., Humphreys, C. J. & Sutton, A. P. Electron-energy-loss Spectra and The Structural Stability of Nickel Oxide: An LSDA+U Study. *Phys. Rev. B* **57**, 1505–1509 (1998).

12. Wang, L., Maxisch, T. & Ceder, G. Oxidation Energies of Transition Metal oxides within the GGA+U framework. *Phys. Rev. B* **73**, 195107–195112 (2006).

13. Aydinol, M. K., Kohan, A. F., Ceder, G., Cho, K. & Joannopoulos, J. Ab initio study of lithium intercalation in metal oxides and metal dichalcogenides. *Phys. Rev. B* **56**, 1354–1365 (1997).

14. Seo, D.-H. *et al.* The Structural and Chemical Origin of the Oxygen Redox Activity in Layered and Cation-disordered Li-excess Cathode Materials. *Nat. Chem.* **8**, 692–697 (2016).

15. Yao, Z., Kim, S., He, J., Hegde, V. I. & Wolverton, C. Interplay of cation and anion redox in Li4Mn2O5 cathode material and prediction of improved Li4(Mn,M)2O5 electrodes for Li-ion batteries. *Sci. Adv.* **4**, eaao6754 (2018).

16. Grindy, S., Meredig, B., Kirklin, S., Saal, J. E. & Wolverton, C. Approaching Chemical Accuracy with Density Functional Calculations: Diatomic Energy Corrections. *Phys. Rev. B* **87**, 075150–075157 (2013).

17. SGTE. *Thermodynamic Properties of Inorganic Materials. vol. 19.* (1999).
