# Supplementary material for: Dynamic imaging of crystalline defects in lithium-manganese oxide electrodes during electrochemical activation to high voltage
Source: Nat Commun. 2019 Apr 12;10:1692. doi: 10.1038/s41467-019-09408-2 (PMC6461632; doi:10.1038/s41467-019-09408-2)
Supplement: Supplementary file 3 — Description of Additional Supplementary Files [file 41467_2019_9408_MOESM3_ESM.pdf]

Description of Additional Supplementary Files

Supplementary Movie 1

The evolution of (001) stacking fault with fault vector of *b*/6[110] Li_2MnO_3 in delithiation observed by in-situ HREM. The frame speed is 64 X faster than real time.

Supplementary Movie 2

In Li_2MnO_3 delithiation, dissociated dislocations with Burgers vector of c/2[001] formed. The frame speed is 2 X faster than real time.

Supplementary Movie 3

In-situ HREM of Li_2MnO_3 in lithiation showing the generation and climbing of the dislocations with Burgers vector of c/2[001]. The frame speed is 8 X faster than real time.

Supplementary Movie 4

In-situ HREM of Li_2MnO_3 in lithiation showing the gliding and climbing of the dislocations with Burgers vector of c/2[001]. The frame speed is 2 X faster than real time.
